# The Hospital Interne1Read at the annual meeting of the Buffalo General Hospital Alumni Association, April 24. 1908.

**Published:** 1908-06

**Authors:** E. L. Shurly

**Affiliations:** Detroit Mich. 32 Adams Avenue, West


### The Hospital Interne.[1]

By E. L, SHURLY, M. D. Detroit Mich.

I DOUBT whether the vast archives of medical history would furnish much literature on the subject of that character of medical life known as the Hospital Interne, or House Physician, or House Surgeon, or Resident Physician, as he may be designated. Yet, such sort of official has been the complement of organised hospitals for at least 400 years, and probably also from earlier antiquity—when the religious temples embraced the foci of medical learning.

However, old as he may be and neglected as he has been, I shall risk censure by taking him for a topic on this occasion, fondly hoping that in the near future I may be justified by the exhumation of his archaic existence, portrayed on a stele as a fine Apollo-like figure beneath a talismanic scroll bearing the word sterility, with a culture tube in one fist, and a rattlesnake in the other, standing on an enbalmed effigy of death, wrapped in iodoform and bichloride gauze, while casting a smile of triumph at a tunicless, aseptic, image of Hygeia by his side who, under the emblem of a trained nurse's cap, holds a scepter bearing the legend, "There is nothing new under the sun,"—but me!

Oh! would that archeology might make this discovery during my lifetime, so that I might be justified in thus assuming to connect such a modern entity as the Hospital Interne with the musty myths of the past. Having passed through the various stages from hospital hangeron, sub-interne, interne, member of hospital staff, and chief of hospital staff, I confess to feeling what might be called a degenerate passion for passing down the gamut to the realm of hospital life—to stand again, a supercilious menial, amidst the activities, the tragedies, and the triumphs of science and art, as encompassed by the sacred walls of a hospital; where is nurtured education, sympathy, courage, ambition, and self-abnegation. What a glorious dog's life it is! where the lashes of censure from many masters whip the sentient nature into self-control, or the balm of encouragement loosens the springs of ardor, hope, and industry. And, where suffering, patience, fortitude, terror, petulance, tragedy, comedy and all human virtues and weaknesses are ever present to excite pity, firmness, philanthropy, and incentive for scientific thought and ethics. What a book for study is thus offered! And what a stack of experience may be thus garnered within those walls.

Sequestered from the outside world as it were, and environed with responsibilities, surprises, dangers, doubts, and censures—variously combining as either balmy showers, or cutting hail

1. Read at the annual meeting of the Buffalo General Hospital Alumni Association, April 24, 1908.

storms,—to foster, or to break the threads of his imperturable self-conceit, or scientific ambition, the Hospital Interne certainly occupies a unique position.

It is true that the outside practising physician also meets with all of these vicissitudes,—even coming with more potent force, because he is acting entirely upon his own responsibility. Yet it is less intense,—more bearable because more diffused over the range of time. Besides, the outside practitioner has no immediate censor to lean upon, or to wield the club of discipline over him. But alas! although so free from outside worry—and so comparatively independent,—the glory of the Interne's progress is frequently shaded by severe and critical reverses, through mistakes, or accidents, or other unfortuitous circumstances.

Among the less fascinating of the disasters which may befall him is that of making a post-mortem examination on the wrong cadaver. Take, for instance, a patient in whom he has been greatly interested during life. At the death he is naturally eager to see the effects of the morbid process which he has watched step by step. He goes to the task of investigation in the dead room, (for the pathologist is seldom there), with a high degree of attention and curiosity. When soon, perhaps, after exposing and unseating the viscera, he fails to find the anticipated lesions—becomes astonished beyond measure, and finally discovers that he has dissected the wrong body. He has, perhaps, mutilated a body which on account of circumstances may bring him, and the institution before the bar of public indignation.

Here, then, is a terrible catastrophe, well-nigh sufficient to convert his scientific enthusiasm immediately into apprehension and remorse; for he is surely in a "bad scrape." He has probably ran hard against a heap of maudlin sentiment, born of barbaric superstition and tradition. In despair and perhaps "rattled" he flies for consolation to his colleagues, and to the solace of his pipe within the sacred precincts of his room, there to plan, and await extrication through the merciful hands of the undertaker.

Nor can we wonder at the surcease of sorrow and the composure which may follow an entrance to this,—a hospital interne's room—his home; when we behold such a spectacle of charming disorder! unequaled by any other particular domiciliary quarters. Come, let us look into one! It contains two beds (to be occupied by four persons perhaps) and is never seen "made up." Two wardrobes, one table, four spittoons, two chairs, one looking glass,—frescoed with the dry remains of splashes of soapy water, or ginger ale; a washstand laden with towels, rags, cracked tumblers, pieces of soap, tooth brushes, cigaret and cigar stubs. Two or four trunks ornamented with bric-a-brac—consisting of human bones, and also fancy embroidered pillows.

One, or perhaps two, bureaus with drawers in various states of adjustment, and with top covers consisting of tobacco begrimed towels, almost hidden from sight by neckties; soiled and clean collars and cuffs; jewelry—principally cuff and collar buttons—photographs, old letters, combs, brushes, surgical instruments, books and circulars. A table covered with a cloth as well stained as any laboratory cloth ever seen, its surface bedecked with about 20 pipes, six boxes of tobacco, empty and partially filled cigaret boxes, four human skulls—collectively containing postage stamps, shirt buttons, rings, string, buttons, playing cards, religious mottoes, and microscopic slides. Cover glasses, pens, pins, pencils, pen-holders, matches, and the like.

There will be on the table also, books, stethoscopes, water-pitchers, surgical instruments, newspapers, magazines, a bottle of black and a bottle of red ink, besides several bottles containing various medicinal mixtures. On the window sill, are shaving mugs, razors, and similar belongings. Photographs, pieces of paper, pieces of pipes, boxes, cigaret holders, memoranda, tumblers, saucers, books, phamphlets, surgical plaster, bandages, ribbons, and the like, while the walls are literally covered in places with college banners, mottoes, cartoons, placques, and photographs of pretty girls and sturdy young men,—interspersed with placards such as "Keep out;" "This is my busy day;" "Go back and sit down;" "Come to Happy Hooligan" and "God bless our home."

And on the floor in and about the room corners, are such personal vestments as soiled underclothing, and linen, shoes, slippers, boxing gloves, books, umbrellas, canes, gowns, baseball bats and the like. In short, the air of elegant disorder hereabout is most typical of man's innate tendency to revolt against the little conventionalities of civilisation. It is however, the interne's Arcadia, although the throne of Choas be installed with mighty grip. I am well aware, however, that this description may not apply to the appearance of the rooms of the modern interne, inas much as the beneficent influence of the trained nurse, undoubtedly exerts a strong restraining influence over man's careless tendencies.

There are at least four classes of hospital internes—namely, the Good, the Self-conceited, the Antomatic, and the Bad. The definitions of these several classes seem hardly necessary, because we have probably been in each class ourselves. I ought, however, to spend a moment in mentioning that the latter class for the development of pure "cussedness" and the extension of its consequences to the staff, their fellows, and the hospital generally, deserves the highest prize which his Satanic majesty can forge. I am glad to say however, that this class numbers but few. The classification mentioned, holds good in whatever manner the members may be selected,—according to my observations. For in one of our large hospitals the selection by examination is practised, while in the other the selection is made by appointment without examination, yet the results are apparently the same. Indeed it often happens that the most scholarly men prove to be the most automatic and inefficient and vice versa.

One cause of the inefficiency of internes may be the short tenure of office, thus making the term of service in the several departments too short. Another, may be the rushing advance of surgery which requires the services of the whole house staff for many hours of the day, and another cause may be the attraction offered by surgery to the young men. The grand direct results, the brilliant effects, the adventurous ante-mortem sorties, all attract the young man's attention and render his interest in a cardiac or other inoperable visceral lesion too tedious, and prosaic.

The interne is often spoiled by members of the medical staff,—some of whom are too careless and lenient; while others are too punctillious. The effect of being on a medical staff works an unfortunate change on some men,—who become austere, fault-finding, and otherwise discomforting. Some are altered by extensive travel,—for instance, a man may go to Europe where the hospitals are supported by State taxation, where labor is cheap and temporal ambition is low, and come back to his little financially struggling hospital, full of new or old ideas, and may undertake to institute all sorts of arbitrary and expensive reforms in discipline, hygiene, or appurtenances,—in accordance with what he has seen abroad. He may thus engender a seige of confusion which is both troublesome and impracticable. Such errors of judgment often confuse, discourage and spoil internes as well as nurses.

The bulldosing of internes and hospital attachés by staff members does not generally secure the best service. However, the behavior of hospital staffs,—especially the surgical members, is not as flagrantly imperious,—if I may be allowed the term,—nowadays, as formerly. Many of us can well remember the insults and the menial life which we led at times. Before the days of the trained nurse the interne fulfilled that important position to a great extent. He was apothecary, wound dresser, orderly, and anesthetist. He gave rectal injections, took all the temperatures, and often attended to the bathing of patients. He was in truth the factotum of the hospital medical administration. He was held responsible for doing, and for not doing all sorts of things.

If he tied the carotid artery to prevent death from hemorrhage on one occasion, he was "blown up" for it, or, if he did not do so on another occasion, he was also "blown up" for not doing it. He would be up all night perhaps with a case of tetanus—administering chloroform, or attending a woman in labor, and then do his routine work the next day just the same; he should not be tired, that were childish. If he did not tie the arteries at an operation, as fast as the surgeon could cut them, he would be insulted right there and then, with no uncertain expletives either,—notwithstanding the third commandment. For surgeons formerly, as now, did not always keep in mind the decalogue. But, notwithstanding all this, hospital work and discipline constitutes a grand, beneficial epoch in one's medical life. The sequestration from outside friction is enchanting, the close contact with suffering humanity and the opportunity for observation of scientific administration for its relief is gratifying. The sieges against disease and death, whether losing or winning, are glorious ones.

To assuage a poor wretch's pain or suffering, or directly save his life, even at the risk of becoming infected is exhilarating. And to work out the precise pathology of the disease during life and even after death is really exalting. Aye! to learn science by study, and art by practice, is a goal worthy of any man's energies, and in hospital life lies the ideal field. To learn and digest what we observe, and to feel what we learn, should be the acme of our ambition. Hospital life, then, should sharpen the faculties, enlarge the senses, develop the reason and broaden convictions. This it may do for the interne, if he takes advantage of his opportunities, and will think hard. Otherwise, it may narrow his mind, cripple his thinking machine. But, besides making him scientific, hospital life may not make of him an efficient outside practitioner among the people. It may take him at least two years to become a good outside practitioner. Why? Because he has been studying organisms only. He has perhaps become too scientific. He has not been studying man as a civic or social being, but unconsciously as an animal only—under discipline. The social and to a certain extent the psychic side of man, he has yet to learn.

His methods and habits in the hospital have been rather laconic and dogmatic. He has not realised the difference between man and a guinea pig. He has not been dealing, so to speak, with man as a free agent, at home. He cannot visit his patient in private practice as he would in the hospital. He cannot go with watch in one hand, and thermometer in the other, making his observations in silence, and brusquely take his leave, with a wave of the hand, and a command to go on the same, or do so and so; ignoring questions, conversation, and the all important suggestions of relatives and neighbors. If he does so, he will be a poor practitioner, in every sense, for he must learn individuality. He must add to his knowledge and expedients—tact. It may be easy to shoot a lion, if one can "get within range," but alas! it may be difficult to get within range.

Neither can scholarship save him from failure always, for people generally care little for erudition which they do not understand. They want relief; they want sympathy; they want congenial touch in addition to scientific effects. These outward and visible signs they must have; and, unfortunately the man without the proper qualities,—whether a scholar or an artist,—can not command the confidence and coöperation of the laity. And when the physician or surgeon with the splendid equipment furnished him by hospital service as an interne, can afterward put himself *en rapport,* equally with the emotional, the superstitious, the ignorant, the intelligent, and the undisciplined elements of society, so as to secure their respect and confidence, he will be able to use successfully his store of science, and his acquisition of art, for the benefit of humanity, and for his own benefit, he will be an esteemed and honored physician and surgeon. He will be able to fulfil the mission so graphically expressed by Stephenson as follows: "The physician is the flower (such as it is) of our civilisation; and when that stage of man is done with, and only remembered to be marvelled at in history, he will be thought to have shared as little as any in the defects of the period, and most notably exhibited the virtues of the race."

32 Adams Avenue, West.

